# Seasonal Land Use and Land Cover Mapping in South American Agricultural Watersheds Using Multisource Remote Sensing: The Case of Cuenca Laguna Merín, Uruguay

**DOI:** 10.3390/s25010228

**Published:** 2025-01-03

**Authors:** Giancarlo Alciaturi, Shimon Wdowinski, María del Pilar García-Rodríguez, Virginia Fernández

**Affiliations:** 1 Programa de Doctorado en Geografía, Facultad de Geografía e Historia, Universidad Complutense de Madrid, 28040 Madrid, Spain; 2 Institute of Environment, Department of Earth and Environment, Florida International University, Miami, FL 33199, USA; shimon.wdowinski@fiu.edu; 3 Departamento de Geografía, Facultad de Geografía e Historia, Universidad Complutense de Madrid, 28040 Madrid, Spain; mpgarcia@ucm.es; 4 Departamento de Geografía, Facultad de Ciencias, Universidad de la República, Montevideo 4225, Uruguay; vivi@fcien.edu.uy

**Keywords:** multisource remote sensing, land use/land cover, Sentinel 1, Sentinel 2

## Abstract

Recent advancements in Earth Observation sensors, improved accessibility to imagery and the development of corresponding processing tools have significantly empowered researchers to extract insights from Multisource Remote Sensing. This study aims to use these technologies for mapping summer and winter Land Use/Land Cover features in Cuenca de la Laguna Merín, Uruguay, while comparing the performance of Random Forests, Support Vector Machines, and Gradient-Boosting Tree classifiers. The materials include Sentinel-2, Sentinel-1 and Shuttle Radar Topography Mission imagery, Google Earth Engine, training and validation datasets and quoted classifiers. The methods involve creating a multisource database, conducting feature importance analysis, developing models, supervised classification and performing accuracy assessments. Results indicate a low significance of microwave inputs relative to optical features. Short-wave infrared bands and transformations such as the Normalised Vegetation Index, Land Surface Water Index and Enhanced Vegetation Index demonstrate the highest importance. Accuracy assessments indicate that performance in mapping various classes is optimal, particularly for rice paddies, which play a vital role in the country’s economy and highlight significant environmental concerns. However, challenges persist in reducing confusion between classes, particularly regarding natural vegetation features versus seasonally flooded vegetation, as well as post-agricultural fields/bare land and herbaceous areas. Random Forests and Gradient-Boosting Trees exhibited superior performance compared to Support Vector Machines. Future research should explore approaches such as Deep Learning and pixel-based and object-based classification integration to address the identified challenges. These initiatives should consider various data combinations, including additional indices and texture metrics derived from the Grey-Level Co-Occurrence Matrix.

## 1. Introduction

Recent advancements in Earth observation sensors, such as improvements in temporal, spectral, spatial and radiometric resolution, combined with the accessibility of Geo Big Data resources, such as artificial intelligence, cloud computing and web applications, have significantly improved the capacity to gain valuable insights into environmental applied science. These technological advancements have made multi-source remote sensing (MSRS) practical for Earth monitoring tasks. MSRS effectively combines the strengths of different observations, addressing the limitations of using a single sensor and offering more accurate and comprehensive monitoring. This approach significantly benefits applications requiring more exhaustive temporal, spatial or spectral information than any single sensor can provide.

Over the past several decades, the focus on MSRS technologies has continued to evolve. For instance, in the 1990s, ref. [1] examined temporal forest cover by utilising Satellite Pour Observation de la Terre (SPOT) and European Remote Sensing Synthetic Aperture Radar–1 (ERS-1) data in a region of South England. Ref. [2] leveraged ERS-1 with a Landsat Thematic Mapper (TM) to monitor vegetation in a semi-arid area of Algeria. Ref. [3] employed TM and the Spaceborne Imaging Radar-C/X-Band Synthetic Aperture Radar to classify geological features effectively. In the 2000s, ref. [4] used ERS-1 and TM to map land use/land cover (LULC) in São Paulo, Brazil. Additionally, ref. [5] combined TM and the European Remote Sensing Synthetic Aperture Radar-2 (ERS-2) to distinguish grassland in eastern Kansas, USA. The authors highlighted encouraging results from the simultaneous application of optical and microwave data for supervised classification.

MSRS supports a variety of applications, including wildfire monitoring [6,7], glaciology [8,9,10], flood monitoring [11,12], soil science [13,14], atmospheric research [15,16], urban planning [17] and LULC monitoring [18,19].

The MSRS is a crucial data source for LULC mapping at regional, national and global levels [20]. Current MSRS data classification approaches could involve labelling pixels using data from different sensors, such as multispectral, hyperspectral, synthetic aperture radar, elevation and light detection and ranging.

LULC classification can be enhanced using additional data sources from citizen science [21,22], social media [23], web scraping [24] and crowdsourcing [25]. Geo Big Data (GeoBD) technologies are essential for managing MSRS data because they enable the retrieval, analysis and processing of large volumes of images from various sources. These technologies provide rapid processing capabilities that result in valuable outputs [26], supported by foundational technologies, such as artificial intelligence and high-performance computing [27]. Open-access platforms, such as Google Earth Engine (GEE) and Microsoft Planetary Computer, play a critical role in facilitating this access, enabling researchers to perform comprehensive analyses and derive insights from extensive environmental data. The LULC scientific literature identifies key targets in agriculture [28,29,30], urban areas [31,32,33], ecology [34,35] and risk management [36,37].

The following cases exemplify notable applications of MSRS. The Sentinel-2 (S2), Planetscope, Digital Elevations Model (DEM) and Canopy Height Model were used for the multiclass categorisation of a complex mangrove ecosystem [38]. TM, Enhanced Thematic Mapper (ETM+) and Operational Land Imager (OLI), with other inputs, were employed to map grasslands [39]. A study [40] classified LULC across six test sites using Sentinel-1 (S1), S2, a geolocated elevation and height metrics product, Visible Infrared Imaging Radiometer Suite, population density data and Dynamic World data. Cropping patterns in the drylands of East Java (Indonesia) were identified using S2, OLI and a Moderate Resolution Imaging Spectroradiometer (MODIS) [41]. Agricultural LULC in Central Java was mapped using the reflective and thermal bands of OLI [42]. The spatio-temporal changes in peatland across various locations in Finland were monitored using ETM+, OLI, Landsat 9, Sentinel-1 (S1) and S2 [43]. These instances illustrate the significant role of global open-data satellite missions in MSRS, whether they rely on multiple platforms or a single platform with various sensors.

**Figure 1:**
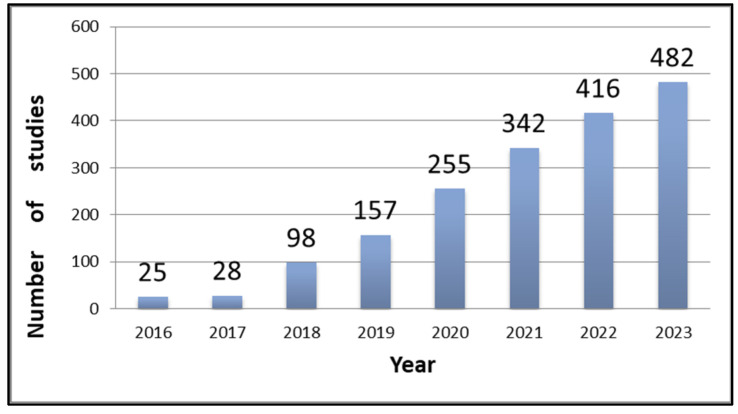
Number of relevant Scopus-indexed studies using Sentinel-1 and Sentinel-2 for remote sensing (2016–2023).

Recent trends [44,45,46] emphasise the importance of programmes led by the National Aeronautics and Space Administration (NASA) and the European Space Agency (ESA). This includes multispectral missions, such as Landsat, Sentinel-2 (S2) and MODIS, as well as radar missions, such as S1. Additionally, DEMs play a crucial role, including data from the Shuttle Radar Topography Mission (SRTM) and the Advanced Spaceborne Thermal Emission and Reflection Radiometer (ASTER). The increasing focus on S1/S2-based MSRS is evidenced by the rise of scientific studies indexed in Scopus between 2016 and 2023 (Figure 1) when the number of documents increased from 25 to 428. This heightened interest may stem from the capability of microwave sensors to effectively differentiate vegetation types by capturing characteristics at the molecular level. Longer wavelengths are especially sensitive to the unique structural features of crops, including the size, shape and arrangement of leaves, stems and water content within the vegetation canopy [47]. These attributes complement the optical mechanisms influenced by the reflectance properties of vegetation, which are determined by factors such as chlorophyll content, leaf structure and water content.

Current S1/S2 LULC mapping may involve diverse approaches, such as traditional classifiers [48,49,50,51] or state-of-the-art deep learning approaches [52,53,54]. LULC mapping may model different timeframes, such as years, seasons, months and days. Currently, most efforts focus on larger temporal scales [55,56], which may not be suitable for specific applications because they overlook seasonally influenced features [57]. These applications may include phenomena such as water quality [58,59,60], land surface temperature [61,62,63], soil biology [64,65,66], soil CO_2_ emissions [67,68], human health [69] and wildlife [70,71].

Recent studies [72,73] highlight the importance of mapping seasonal LULC to support effective natural resource management. However, accurately classifying agricultural features remains a challenge [74] due to seasonal fluctuations in temperature and precipitation affecting vegetation phenology [75], persistent cloud cover and variations in water body surfaces during floods [76].

Regarding effective natural resource management in Uruguay, there is a growing emphasis on examining the relationship between LULC and water quality in various regions, including significant watersheds [77,78], Pampean landscapes [79] and urban areas [80,81]. The Cuenca Laguna Merín watershed (CLM), as a study area, exhibits complex LULC dynamics, featuring diverse natural substrates such as grasslands, native forests (referred to locally as *monte nativo*), wetlands and various land management practices, including cultivated afforestation, artificial pastures and seasonal crops. The region is notably recognised for its extensive summer rice cultivation, which makes Uruguay one of the most important exporters. CLM provides various ecosystem services, including food production, water availability, climate habitability, water quality maintenance, the mitigation of extreme events and the reduction of diseases and pests [82]. Flooded areas are suitable for wetlands that support ecological functions, such as housing a great diversity of flora and fauna [83]. The main socio-environmental factors are wetland contraction and water contamination due to agriculture [84]. The lattermost directly impacts fishing, heritage values and health [85].

As background, technical reports [86,87] employed S2 to map rice paddies and other LULC classes during agricultural years. Focusing on the study area, ref. [88] employed Random Forest to classify six-layer stacks involving S2 and a combination of S1/S2 to map rice paddies and various types of LULC during the summer of 2024 in a test zone covering 5079 km^2^. The most effective alternative utilised a combination of spectral bands, vegetation indices and SAR channels, achieving an overall accuracy of approximately 93% and a Kappa Index of 0.91. Although each SAR feature ranked low in a feature importance analysis, their inclusion as inputs enhanced the classification results compared to approaches that relied solely on S2. This enhancement was particularly evident in differentiating rice paddies and herbaceous crops from other summer crops. Encouraged by this initiative’s outcomes and seeking to improve mapping efforts throughout the entire CLM, this study employs MSRS and GeoBD to fulfil the following objectives:

1. Map LULC for the austral summer of 2024, categorising it into general and specific classes for the season. General categories include herbaceous (HE), cultivated afforestation (CA), native forests (NF), seasonally flooded vegetation (SFV), water bodies (WA) and built-up areas (BU). Seasonal classes comprise rice paddies (RP), other summer crops (OSC) and bare land (BL).
2. Map LULC classes for the austral winter of 2024 using the above-mentioned general categories while incorporating specific classes, such as winter crops (WC) and post-agricultural fields/bare land (PAF).
3. Assess and compare the efficacy of random forest (RF), support vector machine (SVM) and gradient boosting tree (GBT) classifiers.

Advanced classification methodologies, such as deep learning, represent the forefront of technological development; however, these approaches often require components that may not be universally accessible. Consequently, this research focuses on open-access, validated methodologies [89,90,91] to classify multisource S1/S2 data.

This research diverges from previous local experiences because it employs MSRS (containing optical, SAR and elevation data) and optimised classifiers tailored to map LULC for each agricultural season (summer and winter). The main scientific contribution is anticipated to be a pioneering documented experience focusing on seasonal LULC mapping in CLM, a key rice-producing region in South America.

In addition, this research aims to develop tools for analysing the impacts of seasonal agricultural practices on environmental factors such as soil, water and air quality. The resulting maps will facilitate a comparative analysis of the environmental effects of rice cultivation on various LULC features.

## 2. The Study Area

**Figure 2:**
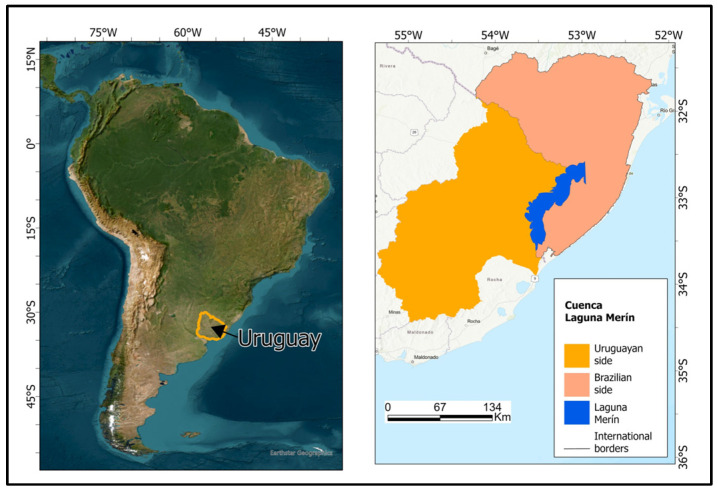
The study area.

Covering an area of 27,892 km^2^, CLM is situated between 31°49′48″ S and 34°26′37″ S, as well as between 53°10′51″ W and 55°21′35″ W. This region forms part of the 62,250 km^2^ transboundary watershed known as Cuenca de la Laguna Merín, which spans Uruguay and Brazil (Figure 2).

The region has a subtropical climate, with an average annual rainfall between 1200 and 1500 mm, mostly occurring in winter [92]. There are noticeable changes in weather throughout the year that affect LULC, particularly due to the varying amounts and strength of rainfall. The area features three types of elevation: mountain ranges (150 to 517 m above sea level), hills (50 to 150 m), and lowlands (below 50 m) [93]. The natural landscape includes gently rolling hills and large plains, with grasslands, wetlands and forest ecosystems [94].

From an agricultural standpoint, the soils in this area do not drain well, but some parts are suitable for mechanised farming. Local streams are important for irrigating rice crops.

## 3. Materials

### 3.1. Multisource Database

The Multisource Database (MSDB) comprises two layers: one for mapping features during the austral summer and another for the winter of 2024. Each layer includes S2 composites, derived indices, S1 composites and SRTM data. The MSDB is produced using Analysis Ready Data (ARD), which ensures that both optical and microwave datasets are pre-processed for consistency in radiometric and geometric attributes. Adhering to these standards is essential for accurately sampling representative spectral values for each LULC class, which are critical inputs for the development of seasonal classifier models, as discussed in Section 4.3.

#### 3.1.1. Sentinel 2: Composites and Derived Indices

The most recent S2 ARD in GEE is the Harmonized Sentinel-2 MultiSpectral Instrument-Level-2A (S2har). This dataset is geometrically rectified and provides bottom-of-atmosphere reflectance through corrections for Rayleigh scattering, atmospheric gases, and aerosol particle absorption and scattering. To create S2 composites, spectral filtering is applied to S2har, including bands B2 (BLUE), B3 (GREEN), B4 (RED), B8 (NIR), B11 (SWIR1) and B12 (SWIR2) from acquisitions with 10% or less cloud cover. Temporal filtering focuses on selecting S2har images that, in addition to identifying general LULC classes, effectively capture the following:

- High vegetative growth and fully mature rice paddies for summer mapping. The suitable acquisition dates were February 1 and March 7.
- Crops in medium to high growth stages for winter mapping. Additionally, it aims to detect recent post-agricultural fields after summer. The suitable acquisition dates were July 25 and August 14.

**Figure 3:**
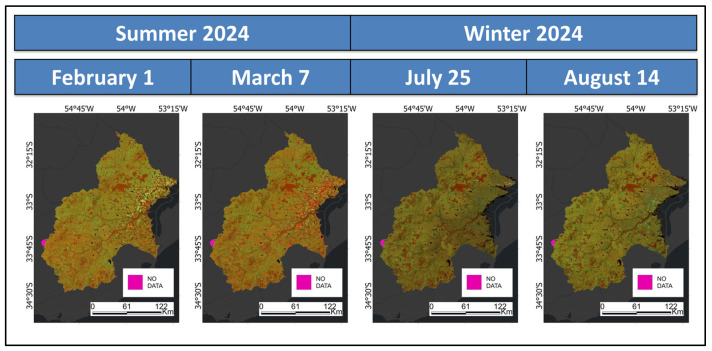
Harmonized Sentinel-2MultiSpectral Instrument—Level-2A composites.

In summary, four S2har composites meet the specified criteria: two from the summer months (February 1 and March 7) and two from the winter months (July 25 and August 14). However, this limited availability restricts using a more extensive time series dataset, which would be preferable for a more comprehensive analysis. In a western flank, the summer imagery reveals a no-data (ND) area of approximately 127 km^2^, while the winter imagery shows an ND surface of about 59 km^2^. Table 1 lists the bands selected for further processing, while Figure 3 shows the RGB composites created using B8, B11 and B4.

**Figure 4:**
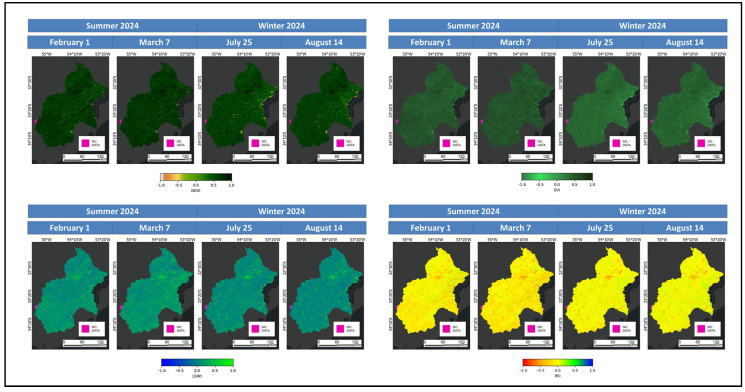
Harmonized Sentinel-2MultiSpectral Instrument-Level-2A indices.

Each S2har composite contains inputs for performing transformations such as the Normalised Difference Vegetation Index (NDVI), Enhanced Vegetation Index (EVI), Land Surface Water Index (LSWI) and Bare Soil Index (BSI). The calculations are computed using the formulas and sources outlined in Table 2. All indices are illustrated in Figure 4.

#### 3.1.2. Sentinel 1 Composites

The S1 products were acquired from the C-band SAR instrument (5.405 GHz) in Interferometric Wide Swath Mode on the descending node, employing Vertical–Horizontal (VH) and Vertical–Vertical (VV) polarisations.

The SAR ARD includes the S1 Ground Range Detected data (S1GRD), incorporating thermal noise, radiometric calibration and SRTM-based terrain correction. The final product is calibrated and ortho-corrected, with terrain-corrected values expressed in decibels (10 × log10(x)), and features both VH and VV channels.

For mapping LULC during the summer, the time filtering was set from 12 January 2024 to 5 March 2024. Acquisitions from 15 July 2024 to 8 September 2024 were utilised for winter mapping. These filtered products are synthesised into medium composites for two consecutive acquisition dates, ensuring comprehensive coverage while minimising temporal variations [99,100,101].

**Figure 5:**
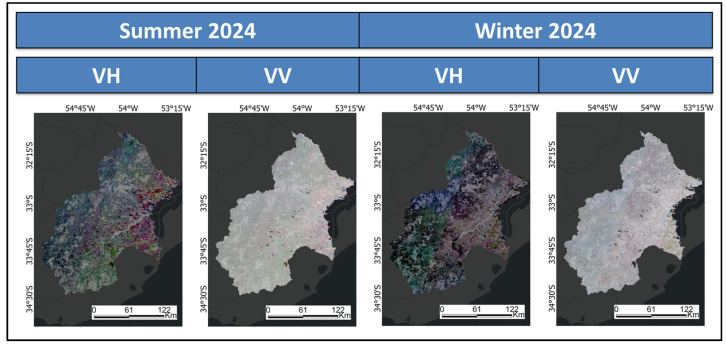
Sentinel-1 Ground Range Detected medium composites per polarisation and season.

Six medium composites per polarisation and season operate as intermediate outputs (Table 3). Four multichannel SAR images are also generated, each showcasing a unique polarisation and season (Figure 5).

#### 3.1.3. Shuttle Radar Topography Mission Digital Elevation Dataset

**Figure 6:**
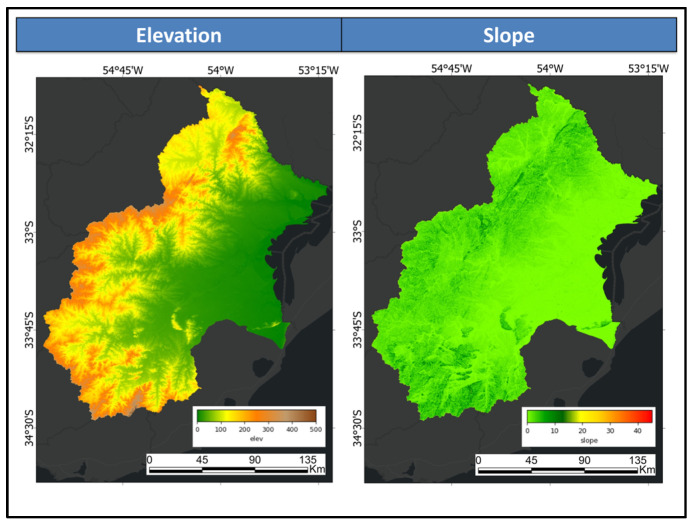
Elevation and slope derived from the shuttle radar topography mission.

The latest version of SRTM (Version 4) was updated to address data gaps. Figure 6 displays elevation data, measured in metres above sea level and slope data, measured in degrees. These inputs aim to enhance the classification accuracy of areas influenced by topography. 

#### 3.1.4. Layer Stacking

The layer stacking systematically organises all composites and indices according to seasonal criteria, resulting in distinct stacks for summer (summer_stack) and winter (winter_stack). Each stack contains SRTM elevation and slope data and comprises thirty-two inputs.

### 3.2. Class Description-Training and Validation Datasets

The subsequent descriptions provide an overview of the scope of each class:

HE consists of natural or artificial pastures and various vegetation types, including herbs, shrubs and trees. This vegetation can remain undisturbed or be influenced by fires or livestock management practices. Bush cover should be at most 25%, while tree cover must be limited to below 10%.

CA pertains to forests cultivated for commercial purposes, such as timber, paper or bioenergy production. These forests are typically situated at higher elevations and on soils unsuitable for agriculture or livestock.

NF refers to natural woodlands that remain untouched by human intervention. Its importance lies in its impact on society, culture and the environment. 

SFVs are often flooded areas with many different types of vegetation. These include shrubs, swamps, woodlands, palm groves and wooded prairies. These can be found in marshy, lacustrine, artificial, riverine and other aquatic systems. Mapping wetlands in the strict sense extends beyond this research’s scope.

WA encompasses rivers, streams and artificial reservoirs for agricultural purposes. The Merín Lagoon is omitted.

BU primarily comprises human-made structures, including buildings, industrial facilities, roads and other infrastructure.

RP refers to areas designated for rice cultivation.

OSC encompasses crops typically sown in late summer and harvested during the same season or in the autumn, such as soybeans and corn.

BL is characterised by minimal or absent vegetation, encompassing landscapes like rocky surfaces, exposed soil, dunes and sandy areas.

PAF includes recently harvested summer crops, primarily rice and soybeans. It also covers the identified areas of scarce and sparse bare land during winter.

WC mainly refers to enhanced pastures for extensive livestock management, which are characterised by more vigorous vegetation than typical herbaceous or different artificial pastures.

**Figure 7:**
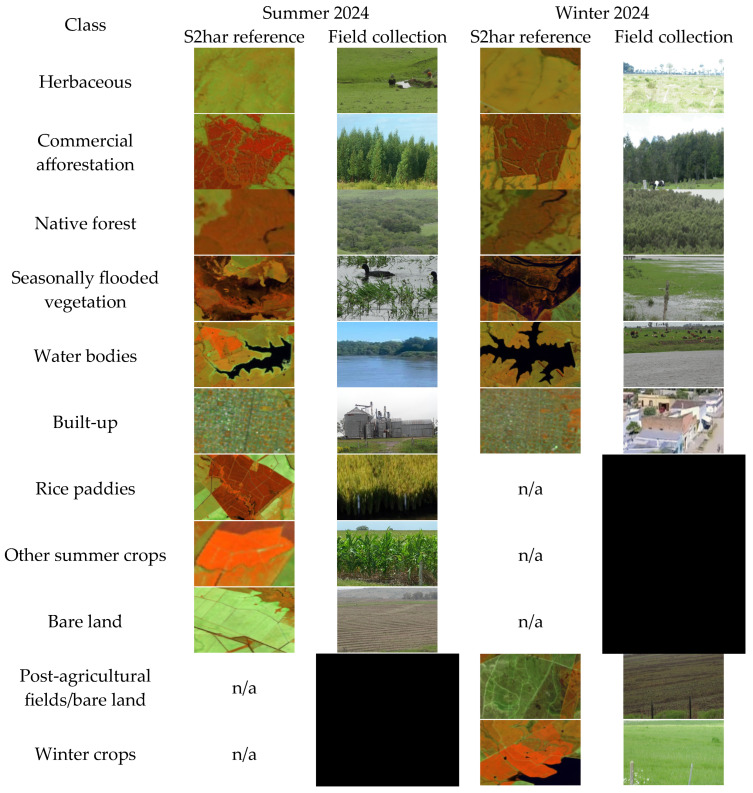
Representative features of each class.

The field collection employed a stratified random sampling approach, adhering to essential principles such as probability sampling and minimising the number of sampling points for cost-effectiveness [102,103]. These points were distributed across various strata to effectively capture the diverse characteristics of the study area. Field data were collected during campaigns on 7–8 March 2024 (for summer cartography) and 3–4 October 2024 (for winter cartography). However, comprehensive spatial coverage during field sampling was hindered by challenges in accessing certain eastern locations, due to limited road networks, private access points within specific crop sites and isolated highland or flooded areas. Notably, the S2har images captured on 7 March and 25 July proved helpful in digitising a limited number of samples that were representative of both summer and winter conditions. This complementary digitisation may influence the accuracy of the SFV, CA and NF classifications. Figure 7 illustrates several examples from each category, including S2har references and field collections.

Table 4 elucidates the distribution of samples (polygons) categorised by season and acquisition approach.

The samples from each season were divided into two separate sets. Half of the samples were used for training, and the other half were used for validation. This method ensured that both sets had about the same number of pixels (Table 5).

Evaluating sampling bias involves methods that are beyond the scope of this research; however, addressing the potential for bias is crucial. Even with skilled interpreters digitising polygons, this process is subject to variability and potential errors [104]. A significant challenge is accurately distinguishing between forested areas [105] and flooded vegetation [106]. These limitations may lead to misclassification, particularly between CA and NF, as well as between SFV and other types of natural vegetation in the eastern part of the study area.

### 3.3. Informatics Resources

Informatics resources fall into two groups: software and classifiers. The software group includes cloud-based services such as GEE, Google Colab, GEEMAP, as well as packages such as Python 3.10 and the SciKit Learn 1.2.2. The classifiers group includes RF, GBT and SVM.

#### 3.3.1. Software

Python 3.10, used in Google Colab, was selected for its compatibility with GEE and the GEEMAP service. GEEMAP integrates seamlessly with Scikit-learn’s 1.2.2 GridSearchCV (GCV) and offers features such as feature importance analysis (FIA) and hyperparameter tuning (HPT).

#### 3.3.2. Classifiers

RF is a robust nonparametric method that employs an ensemble of tree predictors. Each tree within the forest is constructed by independently sampling a random vector from the same distribution for all trees. This versatility makes RF a powerful tool for addressing two- and multi-class classification tasks with large datasets [107]. Furthermore, RF can integrate data from various scales and sources [108]. Three primary hyperparameters (HPTS) must be set to effectively utilise RF: the number of trees, variables per split and the minimum leaf population.

GBT sequentially combines weak learners to minimise errors from previous iterations. Based on decision trees, the approach was enhanced with gradient boosting [109]. This more flexible method refines predictions by iteratively fitting an additive model to the gradient of the loss function residuals [110]. The number of trees, learning rate and maximum features per split are the primary HPTS.

There are key operational differences between RF and GBT. The most notable distinction is that RF grows random decision trees using a random subset of data and features, while GBT constructs decision trees sequentially, guided by boosting [111]. Additionally, RF builds all decision trees in parallel and generates its output as the average of the predictions from these trees. In contrast, GBT builds decision trees one after the other and produces its final output by summing the predictions from all the trees [112]. SVM constructs one or more hyperplanes in high- or infinite-dimensional space to separate data points [113]. The primary objective is to identify the hyperplane that maximises the greatest distance to the nearest data points from any class. Since multiple hyperplanes can separate the classes, the algorithm aims to find the optimal decision boundary by maximising the distance between points [114]. SVM accomplishes this by utilising various kernel functions, such as radial basis function, polynomial, linear, or sigmoid, which assist in mapping the data into a higher-dimensional space where it becomes linearly separable. Grid and cost are the most critical HPTS. 

## 4. Methods

**Figure 8:**
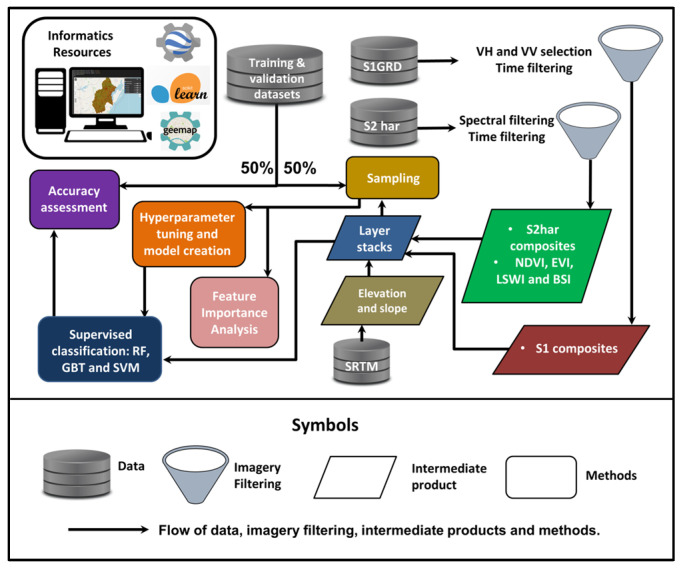
Simplified flow chart of the methodology.

The methods comprise sampling, FIA, HPT, supervised classification and accuracy assessment. Figure 8 shows a simplified illustration of the workflow involving data, imagery filtering, intermediate products (such as composites and indices) and methods.

### 4.1. Sampling

Sampling was conducted using training datasets and layer stacks designed for each season. The sampling results were compiled into two sampling databases: summer (summer_sam) and winter (winter_sam). These databases contain representative features of optical bands, indices and microwave backscattering for each class. These values are essential for conducting FIA and HPT.

### 4.2. Feature Importance Analysis

FIA aims to distinguish the percentage contributions of optical and SAR features in developing RF, SVM and GBT models.

In RF, feature importance is determined by assessing how much each feature contributes to reducing impurity across the ensemble of decision trees. This method relies on Gini impurity metrics. Features that lead to splits that enhance the purity of the nodes are considered highly important. Consequently, features that frequently and effectively contribute to these splits receive higher importance scores [115]. SVM does not use impurity metrics; instead, its objective is to maximise the margin between classes by creating a separating hyperplane. For a linear SVM, feature importance can be evaluated using the coefficients in the decision function. Higher coefficients indicate more significant features [116]. When employing nonlinear kernels, the importance of permutation is an alternative method for assessing feature importance by measuring the decrease in model performance that occurs when each feature is randomly shuffled. In GBT, feature importance is assessed using a method such as impurity reduction but operating within a sequential ensemble framework.

### 4.3. Hyperparameter Tuning and Model Creation

HPT aims to identify the optimal combination of HPTS (models) that enhances the performance of classifiers [117]. This process utilises k-fold cross-validation, a robust method for evaluating model effectiveness [118] and preventing overfitting [119].

Initiatives [120,121] have demonstrated the effectiveness of this approach in selecting suitable models for remote sensing imagery classification. In k-fold cross-validation, the dataset is divided into k mutually exclusive subsets, known as ‘folds’ [122]. In this study, k = 10 is employed. The model is trained iteratively on k-1 of these folds, while the remaining folds serve as the evaluation set. This process is repeated k times, with each fold being used once for testing and k–1 times for training. Table 6 presents the suggested parameter values for developing the model for both summer and winter mapping. The ideal model will be determined using the overall accuracy metric, which is represented as a percentage.

In the context of HPT, the results are organised into summer mapping models, which include SummRF, SummSVM and SummGBT, and winter mapping models, namely WinRF, WinSVM and WinGBT.

### 4.4. Supervised Classification

Each layer stack was classified using the RF, SVM or GBT model. The resulting maps correspond to the name of the deriving model.

### 4.5. Accuracy Assessment

Extensively used statistics (Table 7), such as overall accuracy (OA), user accuracy (UA), producer accuracy (PA) and kappa coefficient (kappa), aided the validation of supervised classification outcomes.

## 5. Results and Discussion

### 5.1. Model Creation

Table 8 indicates that the models achieved optimal performance, suggesting that their implementation of supervised classification may yield favourable results. Additionally, the models developed for summer mapping surpassed those created for winter mapping, highlighting that seasonal differences may affect classification accuracy.

### 5.2. The Importance of Optical and Microwave Features

**Figure 9:**
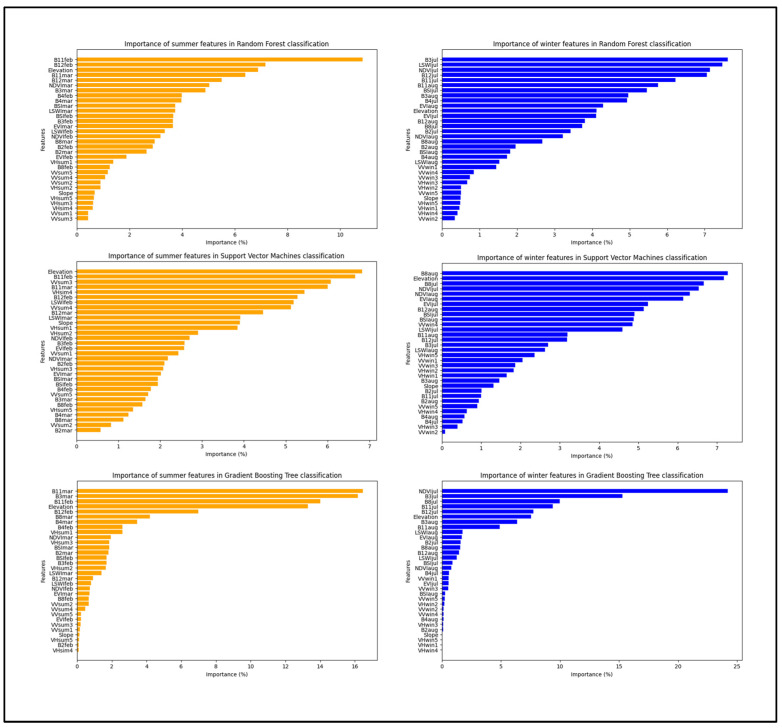
Feature importance according to the feature and the classifier.

The FIA shows clear trends associated with each classifier (Figure 9). RF and GBT, both ensemble methods, exhibit relatively similar patterns due to their use of iterative splits and feature weighting. For instance, optical features consistently demonstrate greater significance than microwave inputs, which aligns with the findings of a recent study about LULC in CLM [88].

SVM focuses on maximising the boundaries between classes. Consequently, it ranks features differently. In this case, microwave features were ranked higher than those from ensemble classifiers. Although SWIR bands usually have lower weights, they remain essential for overall feature ranking.

The dominance of optical features can be attributed to their sensitivity to photosynthetic activity, which enhances their ability to capture patterns of vegetation growth and phenology. General trends show that SWIR bands are significant for summer mapping. For winter mapping, indices such as NDVI and LSWI are especially significant, probably due to the unique characteristics of high soil moisture during winter.

The slightly higher values observed in March indicate that these bands are relevant for capturing phenological changes in vegetation later in the season.

Among the optical bands, B3 was the most relevant in both seasons—particularly in summer for GBT and winter for RF and GBT.

In contrast, B2 and B4 demonstrated moderate and poor importance. The elevation was consistently identified as a significant feature across all analyses, with an importance level ranging from moderate to high. In the SVM analysis, elevation data ranked among the most critical variables, achieving a high importance score.

This research aligns with the findings reported in [123], which indicate that microwave features play a relatively minor role in the analysis. Additionally, it reveals that both VH and VV polarisations hold comparable importance. While SAR features are typically regarded as having low importance—especially in ensemble methods—they demonstrate proven effectiveness in enhancing classification results [88]. Therefore, this research aims to incorporate all available inputs into the forthcoming supervised classifications.

### 5.3. Map Production, Surface Calculation and a Comparison Between Classification Performance

**Figure 10:**
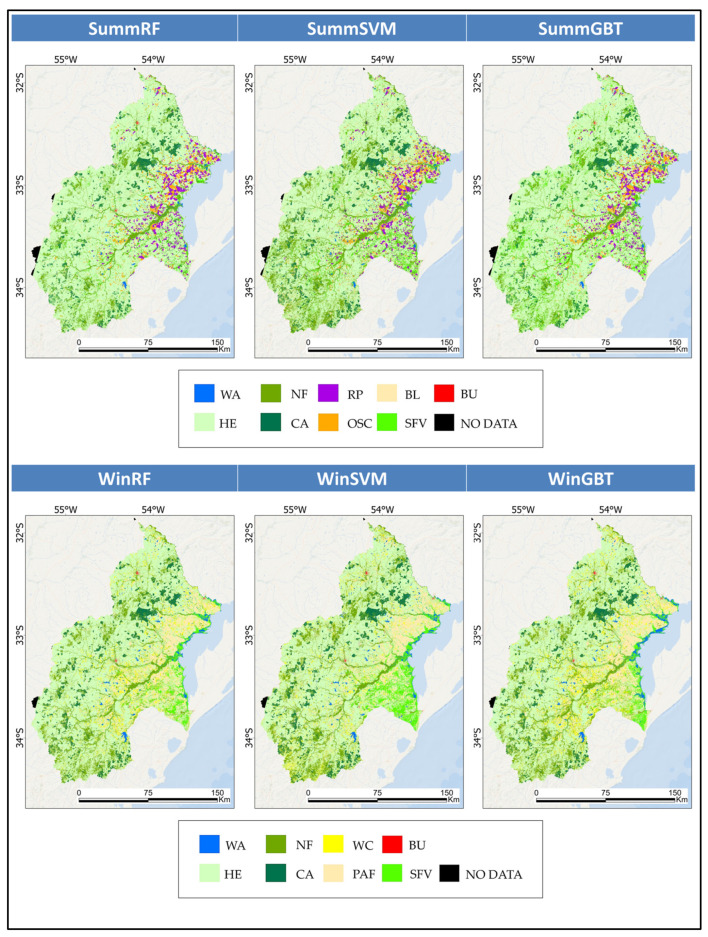
Maps according to the models.

Six maps (Figure 10) were produced from supervised classification, and the corresponding calculated surfaces are outlined in Table 9 (summer) and Table 10 (winter).

Regarding summer cartography, key observations reveal that the HE and NF classes exhibit differences between the methods, with SVM depicting notably lower and higher values. SFV also varies significantly, with GBT having a higher area. Most other classes (WA, CA, RP, OSC, BL and BU) show consistent areas across methods. Overall, RF and GBT tend to produce similar results, while SVM often diverges, especially for HE, NF and SFV.

Regarding winter mapping, notably, HE demonstrates a significantly higher percentage in RF (56.79%) than SVM (52.67%) and GBT (53.37%). In contrast, the SFV class reveals considerable variation, with SVM reporting a much greater area (6.06%) than RF (3.43%) and GBT (3.25%). The WC class also exhibits meaningful divergence, whereas GBT has a higher area (8.02%). Other classes, including NF and BU, maintained consistency across the various methods. Overall, RF and GBT yielded more comparable results, while SVM often diverged, particularly concerning HE, CA and SFV.

To assess the OA and kappa, confusion matrices for the summer (Table A1) and winter (Table A2) maps were generated. In the classifications performed on the summer stack, the RF model achieved an OA of 0.94 and a kappa of 0.92. SVM reached an OA of 0.89 with a kappa of 0.87, while GBT performed similarly to RF, attaining an OA of 0.94 and a kappa of 0.92. For the winter cartography, RF recorded an OA of 0.87 and a kappa of 0.84. SVM matched this OA of 0.87 and kappa of 0.84. In contrast, GBT obtained a slightly better OA of 0.88 and kappa of 0.85.

Table A3 and Table A4 provide the corresponding UA and PA for the summer and winter maps. These statistics help identify the strengths and limitations of classifiers for specific LULC categories.

Regarding summer cartography, the following are key observations:

- RF demonstrates reliable performance across most classes, striking an effective balance between UA and PA. It excels in WA, where it achieves a UA of 1.00 and a PA of 0.96, indicating ideal precision and few omission errors. Similarly, RF performs HE and RP well, showing near-identical UA (~0.95) and PA (~0.96), reflecting its ability to classify these groups accurately with minimal confusion. For CA, RF attains excellent PA, with only minor misclassifications (UA = 0.93). It also performs robustly in NF, achieving a UA of 0.90 and a PA of 0.96, although slight misclassifications lessen precision. RF maintains high accuracies for BL and BU, with UA = 1.00 and PA = 0.96 for BL, as well as UA = 0.92 and PA = 0.89 for BU. However, RF struggles with SFV, whereas PA drops significantly to 0.54, indicating that many actual SFV pixels are missed. A UA of 0.88 displays moderate precision. Despite its challenges with more complex classes, such as SFV, RF remains a strong and reliable classifier for most categories.
- SVM exhibits mixed performance, achieving a high PA for several classes but often struggling with UA due to misclassification errors. SVM performs well in WA, achieving a PA of 0.99 and a UA of 0.97, which means that almost all water pixels are correctly classified, with only minor mislabelling of other classes as water. For NF and BU, SVM achieves very high PA values (0.98 and 0.97, respectively), indicating its ability to correctly identify most actual forest and built-up pixels. However, the UA of 0.88 for NF and 0.80 for BU reveal overclassification issues, whereas other classes are incorrectly labelled as these categories. In HE, SVM struggles more, with a UA of 0.91 and a PA of 0.86, reflecting both omission and commission errors. RP shows a similar imbalance, with a UA of 0.96 but a lower PA of 0.86, meaning that many actual RP pixels are missed. SVM performs the weakest for classes such as OSC and SFV, where both UA and PA values are relatively low (OSC: UA = 0.77, PA = 0.82; SFV: UA = 0.71, PA = 0.64), indicating significant confusion between these and other classes. While SVM excels at detecting certain classes, its overclassification tendency and struggles with complex categories reduce its overall performance.
- GBT is the most balanced and consistent, delivering high accuracy across nearly all classes. It performs well for WA, achieving a UA of 1.00 and a producer accuracy (PA) of 0.92. GBT slightly outperforms RF in HE and CA, achieving a UA of 0.97 and a PA of 0.96 for HE, while maintaining an ideal PA of 1.00 for CA and a high UA of 0.94. For NF, GBT delivers a UA of 0.91 and a PA of 0.96, matching RF in overall reliability and reducing misclassification errors compared to SVM. Notably, GBT exhibits superior performance for classes such as OSC and SFV. OSC achieves a balanced UA of 0.89 and a PA of 0.90. In the case of SFV, GBT outperforms both RF and SVM, with a UA of 0.83 and a PA of 0.65, demonstrating its ability to minimise confusion with similar classes. Additionally, GBT maintains high performance for BU and BL, achieving a UA of 0.92 and a PA of 0.89 for BU as well as a UA of 0.97 and a PA of 0.97 for BL. GBT’s ability to deliver consistently high UA and PA across most classes and its superior performance in handling complex categories, such as SFV, make it the most effective and reliable classifier for summer mapping.

Here are the highlighted comments about winter cartography:

- RF demonstrated strong and reliable performance across most classes, achieving high UA and PA. Notably, RF performs well in WA. In the NF category, it achieved an ideal UA of 0.97 and a PA of 0.98. CA also reflects strong performance, with a UA of 0.91 and a PA of 0.98. RF’s performance in the HE class is moderate, with a UA of 0.79 and a PA of 0.83. Similarly, RF achieves solid but imperfect accuracy for PAF, reaching a UA of 0.84 and a PA of 0.85, indicating a balance between omission and commission errors. For the WC class, RF delivers a UA of 0.89 and a PA of 0.81, performing well in precision but with some underestimation. RF excels in the SFV category, attaining a UA of 0.97 and a moderate PA of 0.78, suggesting high precision but slight underdetection. Lastly, RF achieved excellent results in the BU class, with a UA of 1.00 and a high PA of 0.90.
- SVM delivers variable performance across classes, showing strengths in some categories while facing challenges with UA or PA in others. For WA, SVM achieves an excellent PA of 1.00 but a notably lower UA of 0.69, indicating overclassification. SVM performs well in NF, achieving a UA = 0.98 and a PA = 0.96. In the CA class, SVM achieved an excellent PA of 1.00 and a high UA of 0.94. However, SVM struggles slightly in HE, where it records a UA of 0.86 and a PA of 0.81, indicating a moderate balance between omission and commission errors that is not as consistent as RF. Similarly, SVM achieves balanced performance for PAF with UA = 0.85 and PA = 0.87, showing reliable classification. For the WC class, SVM performed slightly weaker, with UA = 0.85 and PA = 0.78, indicating challenges in identifying all actual WC pixels. In the SFV class, SVM achieved a UA of 0.86 and a PA of 0.78, reflecting moderate performance but lower precision than RF and GBT. Finally, in the BU class, SVM attained a perfect UA of 1.00 and a PA of 0.90, matching RF’s high performance.
- GBT consistently delivers strong, balanced performance across most classes, often matching or slightly outperforming RF and SVM. GBT obtained a PA of 1.00 and a UA of 0.92 for WA, showing slight commission errors vs. RF. GBT excels at NF. In CA, GBT achieves a strong UA (0.97) and PA (0.98), slightly outperforming RF and approaching SVM’s robust PA. For HE, GBT displays a balanced performance, with a UA of 0.82 and a PA of 0.84, surpassing RF but slightly trailing SVM in precision. GBT reliably performs PAF with a UA of 0.85 and a PA of 0.86, matching SVM’s accuracy while maintaining balance. In WC, GBT delivers a UA of 0.83 and a PA of 0.84, showing slightly weaker results than RF but remaining consistent with SVM. Notably, GBT outperforms both RF and SVM in SFV, attaining a UA of 1.00 and a PA of 0.80, indicating a superior ability to minimise misclassification errors while accurately identifying most SFV pixels. Finally, for BU, GBT achieves a UA of 1.00 and a PA of 0.90, matching RF’s and SVM’s performance. Accordingly, GBT emerges as the most effective classifier for winter mapping, while RF remains competitive but illustrates slight weaknesses in complex classes. SVM performs well overall but demonstrates variable precision, particularly for classes that are prone to misclassification.

These results align with recent initiatives [124,125,126,127] demonstrating the adequate performance of RF, SVM and GBT for classifying S1/S2 layer stacks for LULC mapping. Conversely, ref. [128] also reported the influence of classifiers on class-level accuracy. The sampling design introduced bias but did not substantially impact overall accuracy.

### 5.4. Seasonal Changes in Land Use/Land Cover: Overview of Opportunities, Limitations and Prospects

This research does not comprehensively address seasonal LULC changes, as doing so would require methodologies beyond the scope of this study. However, highlighting key summer-to-winter class surface trends is essential because it allows the identification of strengths, limitations and prospects.

The primary strengths can be supported by several factors. CA surfaces exhibit relative stability with minimal fluctuations. The harvested RP, OSC and limited bare land during winter were consistently maintained in PAF. Additionally, BU showed no notable variations.

Two main challenges must be addressed as limitations: the inconsistency observed between the summer and winter surfaces of NF and SFV and the confusion arising from differentiating HE from PAF during winter mapping. The sampling strategy bias discussed in Section 3.2 probably influenced the first challenge. The NF area exhibits higher values in winter than summer across all classifiers. RF and GBT revealed the most significant increases, with RF increasing by approximately 50% and GBT by approximately 72%. In contrast, SVM maintained more consistent NF values between the two seasons, with only a slight distinction of 0.86%. This indicates that SVM may provide more stability for NF classification across seasons, while RF and GBT are more responsive to seasonal changes. Conversely, because this study encompasses natural and artificial forests, misclassifications over NF mapping can be located around CA due to young or sparse cultivated forests between mature plantations [129,130,131]. In addition, forest seasonal spectral responses could vary due to transpiration needs, temperature changes and solar irradiation during summer or winter [132]. Confusion and inconsistencies in SFV are likely due to several factors. These include meteorological conditions and obstructions caused by canopy cover in forested areas [133]. Additionally, variations in water conditions, such as water depth, mixed pixels, organic carbon compounds, water turbidity, chlorophyll content and suspended materials, can contribute to the inconsistencies between natural vegetation categories [134]. The reduction in the HE surface class between consecutive seasons can primarily be attributed to confusion with the PAF class due to their similar spectral behaviour [135,136].

While the advantages of seasonal LULC mapping have been addressed for various instances, large-scale temporal remote sensing mapping remains especially helpful for identifying features such as forests [137,138,139] or seasonally flooded vegetation [140,141].

Current access restrictions to specific sites also limit the complete sampling of the entire CLM, so citizen science initiatives should be promoted as a valuable source for achieving more extensive field sampling across disparate times and locations.

Despite the current advances in MSRS and GeoBD, the outcomes of this research indicate ongoing challenges, particularly with seasonal LULC mapping. The aforementioned challenges match recent review [142] findings. They could be addressed via products with higher spatio-temporal resolution data to capture vegetation changes, comparing remote sensing monitoring methods with direct field observations, comparing remote sensing techniques to ensure accuracy and incorporating seasonal variations and distinctions into phenology extraction models. The scientific community should focus on the latest data availability advancements that enhance MSRS practices. For instance, the Harmonized Landsat and Sentinel-2 project offers a high-resolution, temporally consistent dataset by integrating observations from Landsat and S2. This synthesis enables the frequent and accurate monitoring of LULC dynamics, particularly in regions with high cloud cover and diverse agricultural practices, such as those in CLM.

Research has shown that the Grey-Level co-occurrence matrix derived from S2 and S1 data enhances classification accuracy [143,144]. Therefore, future LULC mapping initiatives in the study area should incorporate textural information. This approach is expected to improve the differentiation of forest areas [145,146], addressing one of the most significant limitations identified in this research. Furthermore, additional initiatives should explore deep learning techniques, which are increasingly utilised in MSRS applications [147]. Compared to traditional pixel-based or object-based methods, deep learning models offer significant advantages, including accumulating knowledge, enhanced generalisation capabilities, improved result quality and the potential to transfer learned models to similar datasets [148,149]. These models can achieve higher accuracy and better generalisation by leveraging the complementary strengths of different data sources and advanced feature extraction techniques [150,151,152]. Quoted deep learning performance may be enriched by innovative feature selection techniques such as a reinforcement learning based the multi-objective differential evolution algorithm [153] or Multimodal Contrastive Learning [154].

### 5.5. Potential Applications of Seasonal LULC Maps

Seasonal LULC mapping has practical applications, especially in exploring how the spatial distribution of usage patterns and substrates affects environmental factors such as air, water and soil quality. For example, a CLM subset encompassing 5583 km^2^ reveals insights derived from Summer and Winter Gradient-Boosting Tree map variations. A notable change observed is the decline in cultivated land, which drops from 969 km^2^ in summer to 384 km^2^ in winter. This may raise inquiries regarding the effects of seasonal fluctuations on different environmental elements such as air, water and soil.

In a study on air quality, ref. [155] employed Sentinel-5P (S5) to analyse the distribution of various pollutants in the region of Gujarat, India. The study specifically examined how LULC influenced the levels of multiple pollutants, including carbon monoxide (CO), sulphur dioxide (SO_2_), nitrogen dioxide (NO_2_), methane (CH_4_) and formaldehyde (HCHO). Furthermore, ref. [156] utilised the S5 to assess air pollutant concentrations of NO_2_, CO and SO_2_ in Jordan from 2019 to 2022. This research revealed that different land cover types produced varying levels of pollution, and each pollutant exhibited seasonal patterns, demonstrating the impact of seasonal activities on air quality.

Human land use activities typically lead to diffuse pollution in water bodies. In contrast, natural vegetation serves to protect water resources by acting as a sink and biofilter [157,158]. Assuming this context, various water quality parameters may be analysed: total nitrogen (TN), nitrates (NO_3_^−^), total phosphorus (TP), phosphates (PO_4_^3^^−^), total suspended solids (TSS), turbidity, biochemical oxygen demand (BOD), chemical oxygen demand (COD), electrical conductivity, pesticides, herbicides, pH, dissolved oxygen (DO) and water temperature.

Research [159,160] reveals that agricultural and urban LULC could negatively impact the physical and chemical quality of soil. These effects involve decreased organic matter, greater soil compaction, and variations in nutrient availability, all of which can undermine soil fertility and disrupt ecosystem functions. Key variables, such as soil moisture, organic matter, temperature, erosion, texture, salinity, compaction, pH, carbon content and mineralogy are particularly important for analysis.

**Figure 11:**
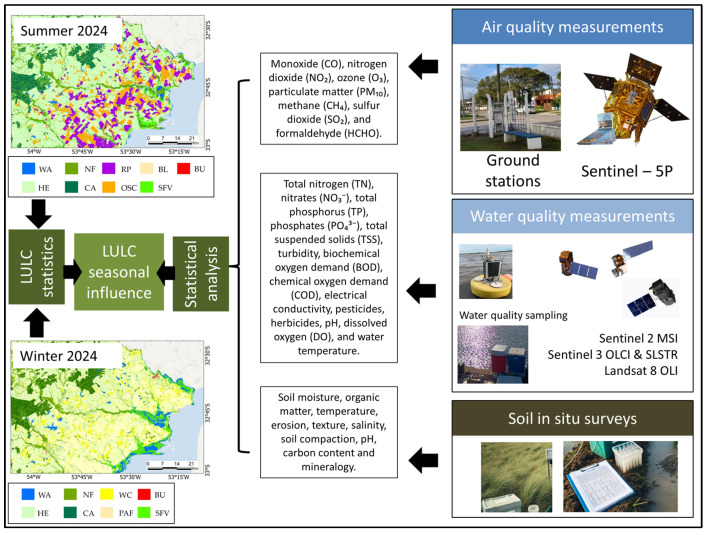
Potential applications of LULC cartography in air, water and soil quality assessments.

Based on the quoted references, it is feasible to suggest that the LULC seasonal component should be considered for agricultural and environmental decision making. Figure 11 exhibits a simplified framework for identifying relationships between LULC and air, water and soil quality.

## 6. Conclusions

This research employed an MSRS approach to map LULC classes within a complex agricultural area during the summer and winter. Seasonal mapping highlights how land management practices and seasonal changes affect the distribution and patterns of LULC. The study utilised a comprehensive array of data types, including optical imagery, SAR and topographic data. The global accuracy metrics and class-specific accuracy indicators generally yielded favourable results, suggesting that the methodologies used were adequate for the CLM. However, comparing the cartographic outputs across the different seasons revealed specific challenges. Notably, inconsistencies were observed in representing forest classes—specifically, NF and CA—in conjunction with the SFV. These classes are often mapped using relatively extensive time series imagery. Despite these limitations, this research lays the groundwork for future improvements in generating more detailed and accurate cartography. Future research on LULC mapping should explore different combinations of the MSRS approach for both summer and winter mapping and prioritise GBT.

These maps can be used in various domains, including agricultural management and environmental protection. For example, mapping seasonal LULC provides valuable insights for the United Nations Framework Convention on Climate Change by highlighting temporal patterns in land cover and agricultural practices. This information is crucial for estimating greenhouse gas emissions related to specific crops—such as rice—and for evaluating the potential for carbon sequestration. Additionally, the cited cartography is critical for analysing LULC structures to enhance ecosystem services and promote coordinated regional development.

## Figures and Tables

**Table 1:** Bands according to composites.

Composites	Bands
February 1	B2feb, B3feb, B4feb, B8feb, B11feb and B12feb
March 7	B2mar, B3mar, B4mar, B8mar, B11mar and B12mar
July 25	B2jul, B3jul, B4jul, B8jul, B11jul and B12jul
August 14	B2aug, B3aug, B4aug, B8aug, B11aug and B12aug

**Table 2:** Indices according to references, formulas and derivatives.

Index	References	Formulas	Derivatives
NDVI	[95]	(NIR + RED)/(NIR − RED)	NDVIfeb, NDVImar, NDVIjul and NDVIaug
EVI	[96]	2.5 × (NIR − RED)/(NIR + 6 × RED − 7.5 × BLUE) + 1)	EVIfeb, EVImar, EVIjul and EVIaug
LSWI	[97]	(NIR − SWIR1)/(NIR + SWIR1)	LSWIfeb, LSWImar, LSWIjul and LSWIaug
BSI	[98]	(SWIR1 + RED) − (NIR + BLUE)/(SWIR 1 + RED) + (NIR + BLUE)	BSIfeb, BSImar, BSIjul and BSIaug

**Table 3:** Medium composites per season, acquisition date and polarisation.

Season	Acquisition Dates	Polarisation	
		VH	VV
Summer	12 January 2024 and 17 January 2024	VHsum1	VVsum1
	24 January 2024 and 29 January 2024	VHsum2	VVsum2
	5 February 2024 and 10 February 2024	VHsum3	VVsum3
	17 February 2024 and 22 February 2024	VHsum4	VVsum4
	29 February 2024 and 5 March 2024	VHsum5	VVsum5
Winter	15 July 2024 and 22 July 2024	VHwin1	VVwin1
	27 July 2024 and 3 August 2024	VHwin2	VVwin2
	8 August 2024 and 15 August 2024	VHwin3	VVwin3
	20 August 2024 and 27 August 2024	VHwin4	VVwin4
	1 September 2024 and 8 September 2024	VHwin5	VVwin5

**Table 4:** Number of samples (polygons) per season and acquisition approach.

Approach	Number of Samples			
	Summer		Winter	
	Amount	%	Amount	%
Field data collection	390	66.32	405	65.75
Visual analysis	198	33.68	211	34.25
Total	588	100	616	100

**Table 5:** Number of pixels for training and validation (similar quantities across different datasets).

	Class											
Season	RP	OSC	HE	CA	NF	SFV	WA	BL	BU	WI	PAF	Total
Summer	579	640	1136	576	812	222	142	256	37	n/a	n/a	4400
Winter	n/a	n/a	1230	497	818	371	196	n/a	63	592	1835	5602

**Table 6:** Classifiers, hyperparameters and proposed values for cross-validation.

Classifier	Hyperparameters	Proposed Values
Random Forest	Number of trees	50, 162, 275, 387, 500
	Min. samples per split	2, 4, 6
	Min. leaf population	2, 4, 6
Gradient Boosting Tree	Number of trees	50, 162, 275, 387, 500
	Learning rate	0.1; 0.2; 1
	Min. leaf population	2, 4, 6
Support Vector Machines	Grid	Linear, polynomial, radial basis function
	Cost	2, 5, 10

Source: adapted from [88].

**Table 7:** Statistics validating supervised classification outcomes.

Statistics	
$OA=\frac{Number\;of\;correctly\;classified\;samples}{Total\;number\;of\;samples}\times 100$	$kappa=\frac{\left(OA-Ae\right)}{\left(1-Ae\right)\;}$ *Ae: the proportion of agreement expected by chance*
$UA=\frac{Number\;of\;correctly\;classified\;samples\;for\;a\;specific\;class}{Total\;number\;of\;samples\;classified\;for\;that\;class}$	
$PA=\frac{Number\;of\;correctly\;classified\;samples\;for\;a\;specific\;class}{Total\;number\;of\;samples\;of\;\;that\;class}$	

**Table 8:** Models according to layer stack, classifier, selected hyperparameters and performance (%).

Model	Layer Stack	Classifier	Selected Hyperparameters	Performance (%)
SummRF	summer_stack	RF	Number of trees = 162 Min. samples per split = 4 Min. leaf population = 2	95
SummSVM		SVM	Grid = lineal Cost = 2	91
SummGBT		GBT	Number of trees = 50 Learning rate = 0.2 Min. leaf population = 2	95
WinRF	winter_stack	RF	Number of trees = 50 Min. samples per split = 2 Min. leaf population = 2	88
WinSVM		SVM	Grid = lineal Cost = 5	88
WinGBT		GBT	Number of trees = 275 Learning rate = 0.2 Min. leaf population = 2	89

**Table 9:** Summer cartography surface according to each model.

	SummRF		SummSVM		SummGBT	
Class	Km^2^	%	Km^2^	%	Km^2^	%
WA	213	0.74	288	1.00	194	0.67
HE	19,817	68.85	17,660	61.36	18,709	65.00
NF	2988	10.38	4574	15.89	2519	8.75
CA	1326	4.61	1428	4.96	1277	4.44
RP	1083	3.76	1034	3.59	1063	3.69
OSC	1059	3.68	1324	4.60	1170	4.06
BL	607	2.11	590	2.05	609	2.12
SFV	1546	5.37	1744	6.06	3103	10.78
BU	16	0.06	14	0.05	12	0.04
ND	127	0.44	127	0.44	127	0.44
TOTAL	28,783	100.00	28,783	100.00	28,783	100.00

**Table 10:** Winter cartography surface according to each model.

	WinRF		WinSVM		WinGBT	
Class	Km^2^	%	Km^2^	%	Km^2^	%
WA	348	1.21	435	1.51	490	1.70
HE	16,347	56.79	15,159	52.67	15,361	53.37
NF	4310	14.98	4326	15.03	4309	14.97
CA	1274	4.43	1462	5.08	1328	4.61
PAF	3644	12.66	4015	13.95	3975	13.81
WC	1795	6.24	1565	5.44	2307	8.02
SFV	988	3.43	1744	6.06	935	3.25
BU	18	0.06	19	0.07	19	0.07
ND	59	0.20	59	0.20	59	0.20
TOTAL	28,783	100.00	28,783	100.00	28,783	100.00

## Data Availability

Data can certainly be provided upon request.

## Appendix A

**Table A1:** Confusion matrices for summer maps.

SummRF										
CLASS	WA	HE	NF	CA	RP	OSC	BL	SFV	BU	Total
WA	136	6	0	0	0	0	0	0	0	142
HE	0	1088	0	0	16	32	0	0	0	1136
NF	0	0	780	31	0	0	0	1	0	812
CA	0	0	0	575	0	0	0	1	0	576
RP	0	0	0	7	556	16	0	0	0	579
OSC	0	27	1	0	16	581	0	15	0	640
BL	0	7	0	0	0	0	246	0	3	256
SFV	0	19	81	3	0	0	0	119	0	222
BU	0	4	0	0	0	0	0	0	33	37
Total	136	1151	862	616	588	629	246	136	36	4400
SummSVM										
CLASS	WA	HE	NF	CA	RP	OSC	BL	SFV	BU	Total
WA	141	0	0	0	0	0	0	1	0	142
HE	2	981	16	0	0	114	0	23	0	1136
NF	0	1	792	17	0	0	0	2	0	812
CA	0	17	3	556	0	0	0	0	0	576
RP	0	0	2	4	496	46	0	31	0	579
OSC	0	72	23	14	3	528	0	0	0	640
BL	0	2	0	0	0	0	245	0	9	256
SFV	3	1	60	0	15	0	0	143	0	222
BU	0	1	0	0	0	0	0	0	36	37
Total	146	1075	896	591	514	688	245	200	45	4400
SummGBT										
CLASS	WA	HE	NF	CA	RP	OSC	BL	SFV	BU	Total
WA	130	4	0	0	1	0	7	0	0	142
HE	0	1087	0	0	16	33	0	0	0	1136
NF	0	0	777	32	0	0	0	3	0	812
CA	0	0	0	575	0	0	0	1	0	576
RP	0	0	0	2	551	16	0	10	0	579
OSC	0	25	13	1	12	574	0	15	0	640
BL	0	5	0	0	0	0	248	0	3	256
SFV	0	0	62	0	0	16	0	144	0	222
BU	0	0	0	0	0	4	0	0	33	37
Total	130	1121	852	610	580	643	255	173	36	4400

**Table A2:** Confusion matrices for winter maps by classifier.

WinRF									
	WA	HE	NF	CA	PAF	WC	SFV	BU	Total
WA	196	0	0	0	0	0	0	0	196
HE	0	1016	13	0	170	26	5	0	1230
NF	0	0	801	0	15	2	0	0	818
CA	0	0	7	487	0	0	3	0	497
PAF	0	242	0	0	1563	30	0	0	1835
WC	0	20	7	0	85	480	0	0	592
SFV	0	1	1	49	30	0	290	0	371
BU	0	0	0	0	6	0	0	57	63
TOTAL	196	1279	829	536	1869	538	298	57	5602
WinSVM									
	WA	HE	NF	CA	PAF	WC	SFV	BU	Total
WA	196	0	0	0	0	0	0	0	196
HE	0	1000	2	0	197	17	14	0	1230
NF	0	1	784	0	9	6	18	0	818
CA	0	0	0	497	0	0	0	0	497
PAF	86	97	0	0	1602	37	13	0	1835
WC	0	64	0	0	66	461	1	0	592
SFV	3	2	17	30	10	20	289	0	371
BU	0	0	0	0	6	0	0	57	63
TOTAL	285	1164	803	527	1890	541	335	57	5602
WinGBT									
	WA	HE	NF	CA	PAF	WC	SFV	BU	Total
WA	196	0	0	0	0	0	0	0	196
HE	0	1034	9	0	153	33	1	0	1230
NF	0	0	794	0	16	8	0	0	818
CA	0	0	9	486	0	2	0	0	497
PAF	4	221	1	0	1575	34	0	0	1835
WC	0	3	0	0	90	499	0	0	592
SFV	12	9	0	16	15	24	295	0	371
BU	0	0	0	0	6	0	0	57	63
TOTAL	212	1267	813	502	1855	600	296	57	5602

**Table A3:** User’s accuracy and producer’s accuracy in summer cartography.

Class	SummRF		SummSVM		SummGBT	
	UA	PA	UA	PA	UA	PA
WA	1	0.96	0.97	0.99	1	0.92
HE	0.95	0.96	0.91	0.86	0.97	0.96
NF	0.90	0.96	0.88	0.98	0.91	0.96
CA	0.93	1	0.94	0.97	0.94	1
RP	0.95	0.96	0.96	0.86	0.95	0.95
OSC	0.92	0.91	0.77	0.82	0.89	0.90
BL	1	0.96	1	0.96	0.97	0.97
SFV	0.88	0.54	0.71	0.64	0.83	0.65
BU	0.92	0.89	0.80	0.97	0.92	0.89

**Table A4:** User’s accuracy and producer’s accuracy in winter cartography.

Class	WinRF		WinSVM		WinGBT	
	UA	PA	UA	PA	UA	PA
WA	1	1	0.69	1	0.92	1
HE	0.79	0.83	0.86	0.81	0.82	0.84
NF	0.97	0.98	0.98	0.96	0.98	0.97
CA	0.91	0.98	0.94	1	0.97	0.98
PAF	0.84	0.85	0.85	0.87	0.85	0.86
WC	0.89	0.81	0.85	0.78	0.83	0.84
SFV	0.97	0.78	0.86	0.78	1	0.80
BU	1	0.90	1	0.90	1	0.90

The following acronyms represent the Land Use and Land Cover (LULC) classes found in Table A1, Table A2, Table A3 and Table A4: Water bodies (WA), Herbaceous (HE), Native forest (NF), Commercial afforestation (CA), Seasonally flooded vegetation (SFV), Built-up (BU), Rice paddies (RP), Other summer crops (OSC), Bare land (BL), Post-agricultural fields/bare land (PAF) and Winter crops (WC).
